# Robotic-Assisted Minimally Invasive Direct Coronary Artery Bypass Grafting with Concomitant Left Atrial Appendage Exclusion

**DOI:** 10.21470/1678-9741-2024-0198

**Published:** 2025-02-14

**Authors:** Gregory Fishberger, Blake Bulard, Leonardo Paim N. da Costa, Lucian Lozonschi

**Affiliations:** 1 Department of Surgery, School of Medicine, University of Colorado, Aurora, Colorado, United States of America.; 2 Division of Cardiothoracic Surgery, Department of Surgery, Morsani College of Medicine, University of South Florida, Tampa, Florida, United States of America.

**Keywords:** Atrial Fibrillation, Coronary Artery Bypass, Echocardiography, Transesophageal, Mortality, Risk, Sternotomy

## Abstract

Off-pump robotic-assisted minimally invasive direct coronary artery bypass (MIDCAB) achieves revascularization without conventional sternotomy and provides benefit to patients that otherwise may not be ideal surgical candidates. For patients with comorbid atrial fibrillation, left atrial appendage exclusion may reduce stroke risk and is achievable via mini thoracotomy during concomitant MIDCAB. Here, we report four patients who underwent off-pump robotic-assisted MIDCAB and concurrent epicardial left atrial appendage exclusion. Intraoperative transesophageal echocardiography confirmed complete left atrial appendage exclusion in all cases. The concomitant robotic approach proved to be feasible, efficacious, and safe, with no postoperative mortality or stroke events during follow-up.

## INTRODUCTION

Coronary artery disease (CAD) and atrial fibrillation (AF) often coexist in patients, as they share common risk factors, and the prevalence of both conditions increases with age^[1]^. Minimally invasive procedures in cardiac surgery have become increasingly utilized in an effort to shorten postoperative recovery and reduce operative morbidity, especially in elderly patients. As such, robotic-assisted minimally invasive direct coronary artery bypass (MIDCAB) enables coronary revascularization through robotic harvesting of the left internal mammary artery (LIMA), followed by anastomosis to the left anterior descending (LAD) artery via direct visualization by left mini thoracotomy. However, this approach is seldom associated with combined procedures due to its high level of complexity. The application of a left atrial appendage (LAA) exclusion device during MIDCAB is uncommon, despite the available evidence supporting that surgical exclusion of the LAA reduces the risk of ischemic stroke and thromboembolic events in patients with AF^[2]^. Therefore, we aimed to assess the feasibility, efficacy, and safety of concomitant LAA exclusion (LAAE) in four patients with known AF undergoing off-pump robotic-assisted MIDCAB.

## CASE PRESENTATION

Between August 2022 and April 2023, four patients underwent off-pump robotic-assisted MIDCABG with concomitant LAAE at our institution. Informed consent was obtained from all patients at the time of surgery.

Patient demographic characteristics are outlined in Table 1. All patients underwent coronary angiography showing severe CAD with hemodynamically significant stenosis within the LAD territory, which was treated with a robotic-assisted MIDCAB of the LIMA to the LAD. Patient D received a hybrid approach and underwent staged percutaneous coronary intervention of the left main to the circumflex artery the following day due to 80% stenosis of the left main coronary artery and 70% mid-LAD stenosis at the time of surgical evaluation.

**Table 1.** Patients’ characteristics.

	Patient A	Patient B	Patient C	Patient D
Age (years)	77	78	77	74
Sex	Male	Male	Male	Male
Smoking status	Never	Never	Former	Current
Preoperative atrial fibrillation	Yes	Yes	Yes	Yes
Preoperative CHF	No	No	Yes	No
Preoperative CHA_2_DS_2_-VASc score	5	4	6	5
Hypertension	Yes	Yes	Yes	Yes
Preoperative T2DM	Yes	No	Yes	No
Preoperative TIA/CVA	No	No	No	Yes, TIA
Preoperative PAD	No	No	No	No
Preoperative COPD	No	No	Yes	Yes
Preoperative CKD	No	No	Yes	No
Procedure time (minutes)	291	299	296	276
LVEF				
Preoperative	61%	60-65%	51%	60-65%
Intraoperative/postoperative	55-60%	60-65%	50%	50-55%
Hospital postoperative LOS	6 days	8 days	6 days	13 days
ICU LOS	3.25 days	4.25 days	1.5 days	2 days
Follow-up since last encounter	17.5 months	15 months	9 months	3 months
Postoperative CVA/TIA	No	No	No	No

CHF=congestive heart failure; CKD=chronic kidney disease; COPD=chronic obstructive pulmonary disease; CVA=cerebrovascular accident; ICU=intensive care unit; LOS=length of stay; LVEF=left ventricular ejection fraction; PAD=peripheral artery disease; T2DM=type 2 diabetes mellitus; TIA=transient ischemic attack

### Surgical Technique

In each case, the patient is placed supine with the left side elevated by a bean bag placed under the mid-torso. The Da Vinci Si system (Intuitive Surgical; Sunnyvale, California, United States of America) is used in all cases. After docking the robot, port access is obtained at the third, fifth, and seventh intercostal spaces (ICS) via trocar placement lined up along the anterior axillary line. The camera trocar is located at the fifth ICS. Instrument trocars are located at the third and seventh ICS. The LIMA is then dissected utilizing the robot platform, in a skeletonized fashion, extending distally until its bifurcation where it is divided between clips. The LIMA is then mobilized, placed at the left lung apex, and the pericardium is opened.

**Fig. 1:**
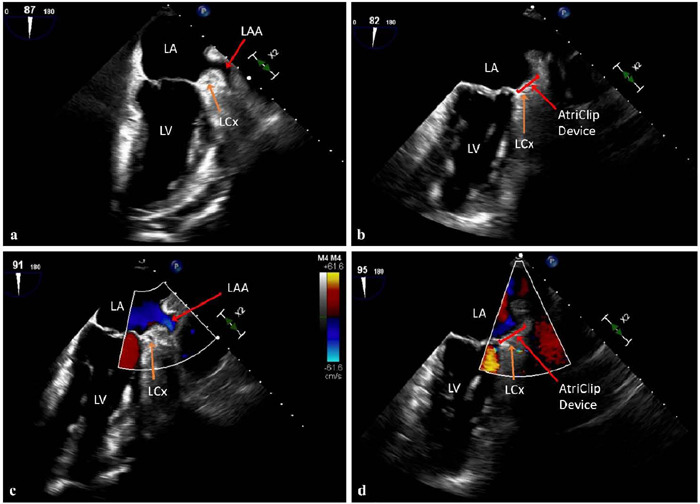
Intraoperative transesophageal echocardiogram with Doppler. Patient D preoperative (panel a) and postoperative (panel b) images following AtriClip application and subsequent left atrial appendage (LAA) exclusion. Complete isolation with no evidence of residual flow into the LAA was confirmed via Doppler mode in preoperative (panel c) and postoperative (panel d) images. LA=left atrium; LCx=left circumflex artery; LV=left ventricle.

The robot is subsequently undocked, and a 7 cm incision is made on the fifth ICS over the anticipated location of the LAD. The Alexis soft tissue wound retractor (Applied Medical; Rancho Santa Margarita, California, United States of America) is inserted followed by a rib spreading device. The opened pericardium is mobilized with silk stay sutures to expose the LAD within the center of the incision. Attention is then driven to the LAA. The LAA is exposed and centered utilizing the off-pump Medtronic Starfish Evo Heart Positioner device (Medtronic; Minneapolis, Minnesota, United States of America) to prevent excess movement of the heart and displace the heart medially for AtriClip placement. An appropriately sized AtriClip Flex-V (AtriCure; Mason, Ohio, United States of America) is positioned at the base of the LAA and deployed under direct vision with additional intraoperative transesophageal echocardiogram (TEE) guidance and visualization. TEE with Doppler confirms complete LAAE, as well as patency of the circumflex artery after clip placement (Figure 1). Lastly, the LIMA is anastomosed to the LAD under direct vision via the pericardial window in a standard off-pump fashion, which utilizes the Medtronic Octopus Nuvo Stabilizer device and 1 mm intracoronary shunt. Graft patency is confirmed via intraoperative transit time flow measurement.

## DISCUSSION

In this case series, we describe our experience with LAA ligation in the context of off-pump robotic-assisted MIDCAB. All patients experienced similar long-term postoperative outcomes with no evidence of cerebrovascular accident, thromboembolism, or mortality during limited follow-up at one year. We confirmed the LAA clip could be applied with minimal modification to the robotic MIDCAB procedure, as the flexible arm of the Flex-V permits angle adjustments to properly reach the base of the LAA. To adequately isolate and expose the LAA, the thoracotomy incision is roughly 2 cm larger than that of standard robotic MIDCAB (7 cm *vs.* 5 cm), and rib spreading is necessary.

The LAA clip was placed after LIMA-LAD anastomosis in patient A; however, for all subsequent patients, it was placed after anterolateral thoracotomy prior to coronary anastomosis. It was found to be more cumbersome to rotate the heart and isolate the LAA following anastomosis due to tethering of the heart with the additional concern of bypass graft injury. Given varying morphology of the LAA, TEE guidance facilitates identification of the appendage neck, orifice, presence of any appendage clot, and complete exclusion. The clip should be placed towards the base of the appendage with care taken to avoid leaving a stump > 10 mm^[3]^. The risks of incomplete exclusion of the neck or excess stump with trabeculated tissue can result in formation of thrombi thus increasing stroke risk^[4]^. Further care should be taken to avoid the inclusion of adjacent epicardial fat and tissue, as well as the neighboring left circumflex artery. Upon clip closure, excess traction can result in kinking of the left circumflex or even occlusion of the artery if the circumflex is entrapped within the device.

The occlusion of the LAA has proven clinical benefit in the literature with significant reductions in short- and long-term stroke incidence^[2]^. Unfortunately, the Left Atrial Appendage Occlusion Study III (or LAAOS III) excluded off-pump and robotic-assisted approaches in the study data. However, it can be presumed that the clinical benefit of complete LAAE would extend to robotic and off-pump cases given the mechanistic origin of most systemic thromboembolic events from the LAA. Additionally, minimally invasive approaches may provide additional benefit to frail or elderly patients who may not be ideal candidates for conventional sternotomy.

Other groups have achieved LAAE in the context of MIDCAB; however, this is the first report to describe combined LAAE in the context of robotic off-pump MIDCAB. Witkowska and Suwalski (2016) described a thoracoscopic approach to LIMA takedown with AtriClip placement^[5]^. Maesen et al.^[6]^ (2020) achieved thoracoscopic pulmonary vein isolation ablation, AtriClip placement, followed by off-pump MIDCAB. Van der Heijden et al.^[7]^ (2022) assessed the efficacy of box-lesion epicardial ablation, LAAE, and off-pump MIDCAB in 23 patients. Antaki et al.^[8]^ (2021) completed 42 isolated robotic-assisted epicardial LAAE. Collectively, these studies demonstrate the absence of stroke in the first year postoperatively. Given that coagulopathy and bleeding diathesis are contraindications to epicardial ablation, LAAE is an ideal strategy for mitigating stroke risk in AF, particularly in patients with contraindications to anticoagulation. Therefore, LAAE in off-pump MIDCAB patients may represent a solution for higher-risk patients with AF and LAD disease. Further studies with larger patient cohorts and extended follow-up are necessary.

## CONCLUSION

In conclusion, LAAE with the AtriClip Flex-V device in patients with AF undergoing robotic-assisted MIDCAB with associated TEE guidance is feasible, efficacious, and safe. Given that LAA clip placement can be achieved with minimal modification to the robotic MIDCAB procedure, patients with a history of AF undergoing MIDCAB should be considered for LAAE.

## Abbreviations, Acronyms & Symbols

**AF** =: **Atrial fibrillation**
**CAD** =: **Coronary artery disease**
**CHF** =: **Congestive heart failure**
**CKD** =: **Chronic kidney disease**
**COPD** =: **Chronic obstructive pulmonary disease**
**CVA** =: **Cerebrovascular accident**
**ICS** =: **Intercostal spaces**
**ICU** =: **Intensive care unit**
**LA** =: **Left atrium**
**LAA** =: **Left atrial appendage**
**LAAE** =: **Left atrial appendage exclusion**
**LAD** =: **Left anterior descending**
**LCx** =: **Left circumflex artery**
**LIMA** =: **Left internal mammary artery**
**LOS** =: **Length of stay**
**LV** =: **Left ventricle**
**LVEF** =: **Left ventricular ejection fraction**
**MIDCAB** =: **Minimally invasive direct coronary artery bypass**
**PAD** =: **Peripheral artery disease**
**T2DM** =: **Type 2 diabetes mellitus**
**TEE** =: **Transesophageal echocardiogram**
**TIA** =: **Transient ischemic event**
